# Relationship quality and exhibitors’ sustainable willingness to participate in exhibitions: A sociocultural perspective

**DOI:** 10.3389/fpsyg.2022.949625

**Published:** 2022-08-24

**Authors:** Pengfei Wang, Liuke Liang, Yu Pan, Yu Wang, Linna Li, Yanyan Chen, Yihan Tian

**Affiliations:** ^1^School of Land and Tourism, Luoyang Normal University, Luoyang, China; ^2^School of Geography and Environment, Henan University, Kaifeng, China; ^3^School of Business, Shanghai University of Finance and Economics, Shanghai, China; ^4^School of Economics and Management, Inner Mongolia University, Hohhot, China; ^5^School of Business, Central South University, Changsha, China; ^6^School of Accounting and Finance, University of Bristol, Bristol, United Kingdom

**Keywords:** exhibitors, relationship quality, guanxi, willingness in sustainable participation, China

## Abstract

Exhibition is a new economic business form in China. From a stakeholder perspective, all important roles in the context of hospitality and tourism are talents. From a socio-cultural perspective, interpersonal relationship quality plays a vital role in business and industrial operations. This constructs a causal model of relationship quality between exhibitors and exhibition organizers, and examined its influence on exhibitors’ willingness to sustainably participate in exhibitions. Data collected from a total of 251 exhibitors from 4 nation-representative exhibitions in Beijing were analyzed. The results found that the four antecedent variables—guanxi (as distinctive from relationship quality), service quality, communication, and exhibition effectiveness—have significant impacts on the relationship quality between exhibitors and exhibition organizers, which in turn, affect exhibitors’ willingness to participate in subsequent exhibitions (i.e., exhibitor loyalty). These findings provide a theoretical basis and policy recommendations talent management in exhibition context.

## Introduction

From a stakeholder perspective, all important roles in the context of hospitality and tourism are talents to be cared and managed. China is a typical human society, and guanxi plays an important role in various social and economic affairs. The past 40 years have been a golden period of rapid development in China’s exhibition industry. According to statistics from the “China Exhibition Economic Development Report (2019),” 3547 economic and trade exhibitions were held nationwide in 2019, with a net exhibition area of more than 13.48 million square meters. The exhibition industry’s rapid development has made competition in the industry unprecedentedly fierce (Hultsman, 2001; Jin and Weber, 2016; Yang et al., 2020; Zhang and Chen, 2020). Establishing and maintaining good relationships with exhibitors and transforming them into loyal customers has become an urgent topic for exhibition organizers (Xu et al., 2007; Son and Jeon, 2012; Jin and Weber, 2013; Wang et al., 2017).

The earliest research on the dimension of relationship quality was put forward by Crosby et al. (1990) on the staff and customers of the life insurance industry, The author proposes that satisfaction and trust are internal variables that constitute relationship quality, while similarity between staff and customers, service skills, and sales behavior are external and pre-variable variables of relationship quality, the improvement of sales performance and the future participation of customers are the outcome variables of relationship quality. Hennig-Thurau and Klee (1997) divided the dimensions of relationship quality into three dimensions: overall quality perception, trust and commitment, and further believed that relationship quality is a mediating variable for customer satisfaction and customer repeat purchases. When Woo and Ennew (2004) studied the conceptual model of relationship quality in the context of B2B, they empirically tested the positive effects of relationship quality on service quality, customer satisfaction, and repeated purchase behavior. Although scholars have not yet reached a consistent conclusion on the dimensional composition of relationship quality, the positive effect of relationship quality on service quality improvement and repetitive purchase intentions has been unanimously recognized by scholars.

The theoretical exploration and empirical research in the field of relationship quality have helped uncover the influence of the relationship between exhibitors and exhibition organizers on willingness to continue participating in exhibitions (Crosby et al., 1990; Hennig-Thurau and Klee, 1997; Jin et al., 2012; Bruwer, 2014; Rodríguez et al., 2015). Nevertheless, compared with the booming exhibition industry, the academic research in this field is obviously insufficient (Jin and Weber, 2016; Jha et al., 2019). Domestic scholars’ early research on the exhibition industry mainly focused on the development status and countermeasures of the exhibition industry, industrial relevance, and socio-economic benefits and influence. Research on the market characteristics of the exhibition industry, exhibitors, audiences and other micro fields is a relative latecomer. The research of foreign scholars on exhibitions is mainly concentrated on microscopic areas such as exhibitors, visitors and exhibitors. On the whole, domestic and foreign scholars still lack research on the quality of the relationship between exhibitors and exhibition organizers.

Although Chinese society is typical of human society, the influencing factors of business performance in China will be quite different from those in Western countries, with strong collectivistic values and high long-term orientation (Wa, 1989; Qin and Li, 2020). The importance of personal relationships in business in developing countries has been widely discussed (Fox, 1987). In China, personal relationships and interpersonal connections are called guanxi, which serves as a substitute for formal institutional support (Xin and Pearce, 1996). As a social exchange mechanism built on mutual favors, guanxi is considered an important strategic asset by most Chinese people and businesses (Park and Luo, 2001; Chu et al., 2019). Moreover, unlike Western countries’ market economy, China’s exhibition organizers generally have a government or industry association background. It poses an unavoidable practical problem within the development of China’s exhibition industry. Liu and Yao (2005) pointed out that the research on relationship quality should be adjusted according to the sociocultural background needs. Combining cultural background information with Chinese characteristics to construct evaluation models for relationship quality is an urgent research task. Therefore, this article selects the relationship quality between exhibitors and exhibition organizers as the research object and discusses the influencing and result factors that affect their relationship quality in the Chinese scenario. This article aims to address theoretical deficiencies in this area and to provide targeted opinions and suggestions for exhibitors to reduce marketing costs and cultivate loyal customers.

There is a lack of research on how one exhibitor influences another willingness to engage in continuous participation in conducting joint events in Chinese and international contexts. Besides, little is known about the mechanism that take place during the process to influence the willingness of the second party. This article is based on professionals interviews and on-site survey investigation to find that what are the important elements that influence the willingness to engage in continuous participation in conducting exhibitions between different exhibitors.

This study contributes to the literature in many ways: first, identify the dimensions of relationship quality in the Chinese context that influence willingness to engage in an exhibition. We incorporate Guanxi as a dimension of relationship quality. The guanxi dimension of relationship quality in this study includes two levels: first, the personal relationship, and second, the business relationship between the enterprise and the government. Second, this article provides an in-depth discussion of the relationship between exhibitors and their willingness to participate in subsequent exhibitions. Third, this paper constructs a theoretical model in which antecedent variables such as service quality, communication, exhibition products, and Guanxi ultimately influence the outcome variables. For instance, such as word-of-mouth and willingness to continue participation through the mediating effect of relationship quality. On this basis, the mechanism of antecedent variables affect the willingness to participate in subsequent exhibitions through relationship quality is tested by empirical analysis. Different from the limitations of previous quantitative research, this article conducts qualitative research during the construction of the relationship between exhibitors and exhibition organizers and designs questionnaires and conducts in-depth interviews with experienced exhibitors and industry experts. On this basis, a quantitative analysis of the data obtained by the questionnaire is conducted. We use a combination of qualitative and quantitative research to ensure the rationality and robustness of our research conclusions.

## Literature review and hypotheses

### Relationship quality theory

The research on relationship quality stems from the rise of relationship marketing theory. Crosby proposed the concept of relationship quality in 1990. Crosby et al. (1990) used sales staff and customers in the life insurance industry as the research objects. From the perspective of interpersonal relationships, they emphasized the importance of sales staff to relationship quality. Relationship quality between the salesperson and the customer represents the degree to which customers depend on salesperson’s honesty, trustworthiness, and past experience. Researchers have explored and generalized Crosby’s findings. However, research on relationship quality mostly concentrated B2C (Business-to-Customer) and B2B (Business-to-Business) segments (Crosby et al., 1990). Scholars are highly interested in the factors/elements that affect relationship quality (Holmlund, 2001; Jin et al., 2012). Over time, researchers focused on identifying the antecedent and outcome variables of relationship quality (Hennig-Thurau and Klee, 1997) and their relationship with service quality (Bruwer, 2014), relationship value and customer loyalty (Huntley, 2006; Rauyruen and Miller, 2007), and the construction of conceptual models that link them logically. Table 1 shows the relationship quality variables identified by the scholars.

**TABLE 1:** List of selected representative research dimensions of relationship quality.

Authors	Antecedent variables	Measurement dimension	Outcome variable
Crosby et al., 1990	Similarity, service skills, sales behavior	Trust and satisfaction	Sales performance continued purchasing
Morgan and Hunt, 1994		Trust and commitment	
Hennig-Thurau and Klee, 1997	Customer satisfaction	Overall quality perception, trust and commitment	Continued purchasing
Fletcher et al., 2000		Satisfaction, commitment, intimacy, trust, passion and love	
Woo and Ennew, 2004		Corporate cooperation, adaptability and environmental atmosphere	Service quality, customer satisfaction and repeat purchases
Gounaris, 2005	Customer orientation, relationship orientation and service provision	Satisfaction and trust	Continued purchasing, relationship maintenance and word of mouth
Huntley, 2006		Technical dimension, social dimension, economic dimension, time dimension, goal alignment, commitment and trust	Sales performance and word-of-mouth publicity
Kijewski et al., 1993		Service quality, trust, satisfaction and commitment	Repeat purchases and loyal attitude
Vieira, 2009	Communication	Customer-centric, relationship net benefits, common goals and commitments	Customer loyalty
Jin et al., 2012		Service quality and relationship satisfaction, trust and emotional commitment, communication, calculable commitment	

### Exhibitors and organizers

Exhibitors and organizers are the most important stakeholders in any exhibition. From the perspective of exhibitors, previous studies focused on participation decisions (Kijewski et al., 1993), participation motivation (Tanner et al., 2001), participation behavior (Huang et al., 2020), and participation performance evaluation (Juhee and Thomas, 2008). Studies on exhibitions organizers are primarily concerned with comparisons of the competitiveness of urban exhibitions (Kang et al., 2005; Aditya, 2019) and the choice of exhibition destinations (Kang et al., 2005). Australian scholars (Jin et al., 2012) examined related articles published in international journals and noted that the current research on exhibitions mainly focuses on exhibition selection, performance, goals, behavior, satisfaction, and on-site service. There are great deficiencies in the research on exhibitors relationship in terms of quality, economic benefits, and impact.

With an average annual growth rate of 20%, China’s exhibitions industry has attracted researchers, policymakers, and investors to research Chinese exhibitors and organizers from various aspects. Fletcher et al. (2000) took the exhibitors’ evaluation of the exhibition as its research subject, focusing on surveying exhibitors’ satisfaction. Liu (2006) conducted a detailed study on exhibitors’ consumption behavior. Yang and Lei (2009) investigated the “2007 Shanghai Electronics Industry Exhibition” exhibitors to identify the critical factors affecting exhibitors’ choice of an exhibition and provide effective suggestions for exhibition destinations and exhibitors on how to improve their competitiveness. Luo (2007) conducted research on the factors affecting exhibitors’ participation decision-making and pointed out that organizer reputation, transportation, exhibitor location, exhibition cost, are vital elements that influence exhibitors’ decision-making. Luo and Bao (2007) noted that different exhibition purposes would significantly affect exhibitor performance evaluation.

Literature on organizers and exhibitors has emerged; few studies focused on the relationship between organizers and exhibitors. As evidenced by the current state of research in the field, most studies remain focused on satisfaction and performance of the exhibition (Tanner et al., 2001; Juhee and Thomas, 2008), and there remains a lack of research on the relationship between organizers and exhibitors. Jin et al. (2012) conducted an exploratory study on the quality of relationships between organizers and exhibitors. This research found that the relationship quality between exhibitors and organizers is determined by service quality and relationship satisfaction, trust and emotional commitment, communication, and computable commitment. However, they did not study the mechanism of the relationship between organizers and exhibitors and their willingness to participate in subsequent exhibitions, nor did they include the context of Chinese cultural elements.

### Determination of the dimension of relationship quality

Many scholars consider relationship quality from social interaction perspective (Xin and Pearce, 1996; Park and Luo, 2001; Qin and Li, 2020). When establishing measurement dimensions, researchers often make specific choices based on different industries. For example, in the B2C industry, researchers mostly consider relationship quality from an interpersonal communication perspective, paying more attention to personal characteristics such as sales staff personality and communication skills. In contrast, B2B attention has been paid to marketing channels, relationship costs, relationship benefits, problem-solving ability, communication quality, cooperation, and participation. Different industries identified varying dimensions that influence relationship quality; however, all are agreed on two dimensions, “trust and satisfaction” are more important. Therefore, based on many previous research results, this article takes satisfaction and trust as the dimensions to measure the quality of the relationship between exhibitors and exhibition organizers. The reasons for selection are as follows.

#### Satisfaction

Satisfaction has been regarded as one of the most critical dimensions in the study of relationship quality in relationship marketing (Holmlund, 2001; Hailin and Emily, 2010; Rai and Nayak, 2018). It describes a customer’s emotional state regarding products or services. In relationship quality, satisfaction indicates that the organizers’ products and services have met the exhibitors’ expectations.

#### Trust

There are many ways to interpret trust. We interpret trust as confidence in the reliability and honest character of the partners. The research results of Jin et al. (2012) show that trust is the most critical factor affecting both exhibitors and organizers. Morgan and Hunt (1994) pointed out that trust is the core component of any relationship quality. They interpreted trust as a combination of good intentions, ability, and honesty. In the context of the exhibition industry, the trust of exhibitors in organizers mainly refers to the exhibitors’ trust in the exhibition information provided by the organizer and the organizer’s fulfillment of their commitments.

### Antecedent variable of relationship quality

#### Service quality

Crosby et al. (1990) asserted that service quality is crucial for the smooth completion of transactions. Zeithaml et al. (1988) divided service quality into five dimensions: tangibility, reliability, responsiveness, security, and empathy. Both Xu et al. (2007) and Zhang L. et al. (2010) showed that service quality has an important effect on relationship quality. In the exhibition industry, the service quality of organizers involves three links, which occur before, during, and after the exhibition. Exhibitors’ evaluation of service quality mainly involves whether the organizer responds to and solves problems that arise promptly and has the management capabilities.

#### Communication

Communication mainly refers to the formal or informal sharing of vital information between the two parties. The exchange of information is an essential feature in a business relationship. Communication is considered a critical dimension of relationship quality (Zhang S. X. et al., 2010). Communication is also an important indicator that affects relationship quality between organizers and exhibitors (Kang and Schrier, 2011; Van Niekerk, 2017). The exchanges studied in this article include proactive contact with exhibitors by the organizer, timely notification of changes to the exhibition, and the exchange atmosphere between the two parties.

#### Exhibition effectiveness

According to the “Report on China’s Convention and Exhibition Economic Development,” published by the China Council for the Promotion of International Trade, the quality and quantity of exhibitors and the quality and quantity of visitors are the most important factors affecting satisfaction exhibitors and visitors. Luo and Bao (2007) also pointed out that the quality and quantity of exhibitors and visitors are the most important evaluation factors determining the intrinsic quality of an exhibition product. Good exhibitor quality and quantity and good audience quality and quantity will satisfy both exhibitors and visitors. Satisfaction is one of the most important measurement dimensions of relationship quality; in other words, a good exhibition effectiveness can affect the relationship quality between organizers and exhibitors. Therefore, this article believes that participation is a factor that affects the quality of the relationship between organizers and exhibitors.

#### Guanxi

Guanxi is a unique cultural phenomenon in Chinese society. It emphasizes personal relationships or relationships in “circles” and refers to “face.” The well-known master of Chinese studies, Mr. Liang Shuming, pointed out that China is a typical ethical-based society, so Guanxi exist in all aspects of the society. Guanxi has played a major role in Chinese society throughout its long history (Alston, 1989; Farh et al., 1998). Over the last few decades, the modernization and globalization of the Chinese economy forced multinational companies to look for ways to establish their own guanxi networks in China (Davies et al., 1995; Lee et al., 2018). Liu and Yao (2005) pointed out that research on relationship quality should consider the needs of the sociocultural background. Making appropriate adjustments and combining cultural background information with Chinese characteristics to construct an evaluation model of relationship quality is an urgent research task.

In fact, Chinese interpersonal behavior has rich cultural connotations. Relationship marketing is a natural orientation for Chinese people to infiltrate interpersonal activities into market activities or economic activities. Therefore, China’s marketing is relationship marketing from the beginning (Zhuang and Xi, 2003). Bian and Zhang (2013) believe that, compared with the weak linkage, single function and occasional obligation of western social capital, China’s relational social capital has three characteristics: strong linkage, functional reuse and frequent obligation. In the Internet age, the Internet has given people the possibility to restructure their relationship circle (Peng, 2019).

In the current environment of China’s exhibition industry, the government or industry associations play an important role. According to research data, 80% of the exhibitions held in Beijing had the background of government or industry associations (Liu and Li, 2009; Dyer et al., 2018; Dekker et al., 2019). Therefore, it is also necessary to study the influence of the “Guanxi” of the government or industry associations on relationship quality. This study’s dimension of human relations includes two levels: first, the personal relationship between people, and second, the business relationship between the enterprise and the government or industry association.

### Outcome variable of relationship quality

Good relationship quality can increase customer loyalty, and customer loyalty can also create word-of-mouth publicity. This conclusion has been confirmed in many previous studies (Kim and Cha, 2002; Rauyruen and Miller, 2007; Cao et al., 2009). Therefore, this article selects customer loyalty and word of mouth as the relationship quality variables.

### Research hypotheses and conceptual model construction

Based on the research findings of Gounaris (2005); Kim and Smith (2007), Dong et al. (2012), and Luo (2007) on the antecedent variables affecting relationship quality, we proposed the following hypotheses.

H1 Service quality, communication, the participation effect and guanxi significantly affect the relationship quality between organizers and exhibitors.

H1a Service quality is one of the antecedent variables affecting relationship quality;

H1b Communication is one of the antecedent variables affecting relationship quality;

H1c The exhibition effectiveness is one of the antecedent variables that affect relationship quality.

H1d Guanxi is one of the antecedent variables that affect relationship quality.

The research results of Crosby et al. (1990) show that relationship quality exerts a direct impact on consumers’ repeated purchase behavior (customer loyalty). Hennig-Thurau and Klee (1997); Rauyruen and Miller (2007), and Zhang and Wu (2007) also concluded that relationship quality has a positive effect on customer loyalty in a B2B environment. Woo and Ennew (2004) and Cao et al. (2009) verified that relationship quality significantly impacts consumer loyalty in their empirical analysis of the service industry. Based on this research, we propose the second hypothesis:

H2 The relationship quality between organizers and exhibitors significantly affects customer loyalty and word-of-mouth.

H2a Relationship quality exerts a significant impact on exhibitor loyalty.

H2b Relationship quality exerts a significant impact on word of mouth.

Generally speaking, loyal consumers are more willing to recommend their trusted brands or products to those around them. Especially in services, a positive postpurchase attitude will lead to a positive reputation among consumers. Gounaris (2005) and Cao et al. (2009) conducted an empirical analysis on the relationship between customer loyalty and customer reputation in the catering industry. The results showed that customer loyalty has a positive effect on word of mouth. Some scholars believe that customer word-of-mouth should be the antecedent variable of customer loyalty, and customer loyalty is the outcome variable of customer word-of-mouth. We find a causal relationship between customer loyalty and word of mouth in the results of previous research, but the conclusions are not uniform. In this study, the exhibitor’s reputation is regarded as an outcome variable of exhibitor loyalty for verification. Based on the above research, we obtain the following hypothesis:

H2c The customer loyalty of exhibitors has a positive effect on word of mouth.

**FIGURE 1:**
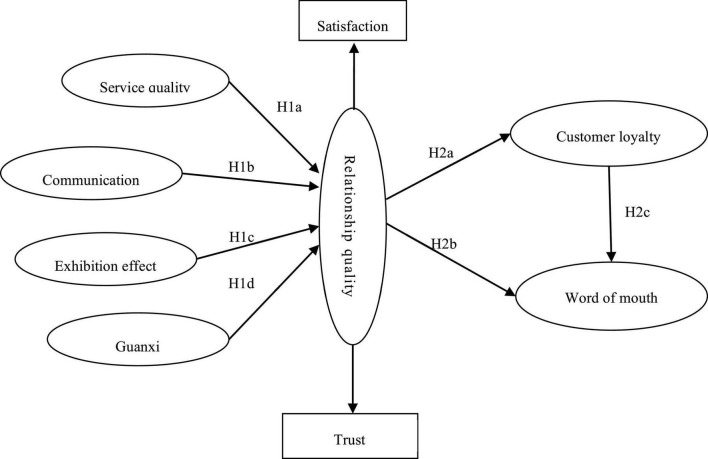
Conceptual model of relationship quality between organizers and exhibitors.

Theory of perceived value holds that consumers’ evaluation of perceived value wouldl be affected by personal factors. Similarly, exhibitors’ perceptions of factors affecting relationship quality be affected by the company’s own characteristics, such as the nature of the company’s assets and company size, location, and exhibition experience (Figure 1).

## Methodology

### In-depth interviews

The author chose the organizers of two exhibitions and eight exhibitors for in-depth interviews. These interviews mainly focused on the factors affecting the relationship quality between exhibitors and exhibition organizers and the importance of relationship quality in the exhibition industry. The interview outline consists of four questions, namely, why the exhibitors want to participate in the exhibition, which factors will affect the exhibitors’ participation decision, how to maintain the quality of the relationship with the organizers, and whether the Guanxi factors will affect the participation decision.

The results of the interview illuminated certain characteristics particular to the exhibition industry: First, even if the effect of this exhibition does not meet exhibitor expectations, as long as the quality of the relationship between the organizer and the exhibitor is in good state, the exhibitor will not refuse to participate in the next exhibition; this characteristic of exhibitors highlights the importance of relationship quality in the exhibition industry. Second, trust is the most critical factor for exhibitors in evaluating the quality of their relationship with organizers. the organizer’s commitment to the exhibitor must be fulfilled, and all information provided by the organizer must be true. These two elements are the most critical part of the exhibitor’s evaluation of the quality of the relationship with the organizer. Third, the quality and quantity of exhibitors and professional visitors are important factors affecting exhibitors’ satisfaction. Fourth, China’s exhibition industry has developed and emerged under government leadership. Compared with other countries and regions, the government has a deeper involvement and a greater role in the exhibition industry. Therefore, for exhibitors, exhibitions organized by the government (or industry associations) or exhibitions to which they are invited by the government (or industry associations) are more likely to attract participation. These findings served as an important reference value for the design of this questionnaire. After our literature search and in-depth interviews, we initially designed a relationship quality measurement scale consisting of 24 options.

### Measurement

The antecedent variables of relationship quality are divided into four aspects: service quality, communication, exhibition effectiveness, and guanxi; the measurement dimension is composed of satisfaction and trust; the result variables are customer loyalty and word of mouth. The questionnaire survey design is based on the Table 2 and it was divided into four parts. Service quality, communication, exhibition effectiveness, guanxi, satisfaction, trust, customer loyalty and word of mouth is mearsured by Likert 5-point scale, from “strongly disagree (1)” to “strongly agree (5).” To facilitate subsequent writing and calculation, the options contained in each part are marked with x1, x2, etc. To improve and modify the questionnaire, the author contacted five professors from Beijing International Studies University, Sun Yat-sen University, and the Chinese Academy of Social Sciences through interviews, phone calls or emails, asking them for their opinions on model construction and questionnaire revision. After modification by these experts, we deleted two of the 24 variables and merged the other two factors into one variable. Therefore, the final relationship quality scale is composed of seven dimensions and 21 variables.

**TABLE 2:** Definition of variables affecting relationship quality, with their sources.

Dimension	Sources
Service quality	Zhang L. et al., 2010; Jin et al., 2012; Wang et al., 2017
Communication	Vieira, 2009; Zhang S. X. et al., 2010; Jin and Weber, 2013; Jin and Weber, 2016
Exhibition effectiveness	Luo and Bao, 2007; Jin et al., 2012; Möller and Halinen, 2017
Guanxi	Alston, 1989; Davies et al., 1995; Farh et al., 1998; Liu and Yao, 2005; Dong and Yang, 2006; Dong et al., 2012; Lee et al., 2018; Qin and Li, 2020
Satisfaction	Crosby et al., 1990; Fletcher et al., 2000; Liu and Yao, 2005; Rauyruen and Miller, 2007; Cao et al., 2009; Jin et al., 2012; Zhang and Chen, 2020
Trust	Hennig-Thurau and Klee, 1997; Fletcher et al., 2000; He, 2006; Rauyruen and Miller, 2007; Wen and Ning, 2008; Liu and Li, 2009; Jin and Weber, 2013; Rodríguez et al., 2015; Jha et al., 2019
Customer loyalty	Crosby et al., 1990; Huntley, 2006; Rauyruen and Miller, 2007; Cao et al., 2009; Dong et al., 2012; Rai and Nayak, 2018
Word of mouth	Gounaris, 2005; Huntley, 2006; Cao et al., 2009; Jin et al., 2012; Wang et al., 2017

Source: Compiled by the author.

To clarify the language of the questionnaire to make it more understandable and express the meaning more effectively, this article selected two exhibitions held in Beijing for on-site survey investigation. The options and language expression of the questionnaire were revised according to the feedback and revision suggestions obtained from the pre-issued questionnaire.

### Data collection

The participants of this research included exhibitors. The authors took the four exhibitions held in Beijing as the research target: the “Beijing International Cultural and Creative Expo,” the “Beijing Tourism Commodity Fair,” the “Beijing Boutique New Year Fair” and the “Beijing International Bright Jewellery Exhibition.” The author randomly conducted one-to-one questionnaire surveys from exhibitors. A total of 400 questionnaires were distributed in this survey; 309 questionnaires were returned, and 251 valid questionnaires were identified. The distribution of valid samples is shown in Table 3. The investigators participating in this research task are all experienced graduate students, most of whom are majoring in convention and exhibition, with the rest majoring in tourism management.

**TABLE 3:** Questionnaire information.

Name	Research location	Sample distribution	Valid sample	Proportion (%)
Beijing International Cultural and Creative Expo	China International Trade Exhibition Center	200	148	60.0
Beijing Tourism Commodities Fair	China International Trade Exhibition Center	100	56	22.3
Beijing Boutique New Year Fair	China International Trade Exhibition Center	50	23	9.2
Beijing International Bright Jewellery Exhibition	China International Trade Exhibition Center	50	24	9.5
Total		400	251	100

The China International Exhibition Center mentioned in this article is the Sanyuanqiao Old National Exhibition Centre.

### Measure reliability and validity analysis

#### Sample reliability test

In this study, Cronbach’s alpha coefficient was used to detect the internal consistency of the selected samples in the measurement options. The results show that the alpha coefficient value is 0.940 (greater than 0.9), indicating that the scale of this study is highly reliable in detecting factors such as the prevariable and outcome variables of the relationship between exhibitors and organizers.

##### Sample size

In SEM analysis, the optimal number of samples has not yet been determined (Wu, 2010). Pu et al. (2019) suggested that if the measured variable has a normal or elliptical distribution, five samples for each observed variable can be adopted; DiPietro et al. (2008) asserted that there is no absolute correlation between the number of samples and the number of observed variables and that the number of samples should be no less than 150. There are 21 explicit variables observed in this paper, and the number of effective samples collected is 251. According to the judgment standards domestically and abroad, the number of samples in this article meets the relevant requirements.

##### Normal distribution test

In this paper, the most commonly used maximum likelihood method used by scholars will be used in the AMOS analysis, which requires the tested variables to conform to the assumption of normal distribution. The results show that, the significance level (*P*-value) of the normal distribution test for the 21 tested options is less than 0.05. Therefore, the scale of this questionnaire passes the normality hypothesis test.

##### Reliability test of latent variables

One of the main purposes of this article is to verify the path relationship between latent variables and the reliability test of latent variables is particularly important. As indicated by the results in Table 4, the reliability test values (Cronbach’s α) of all latent variables exceed 0.8, indicating that the reliability of the latent variables of this questionnaire is high (Fornell and Larcker, 1981). In summary, the data collected in this article are suitable for SEM analysis. From the results of factor load, the values of all variables are between 0.612 and 0.889, which meets the standard of 0.50–0.95 (Pan and Liu, 2018). The combined reliability (CR) of dimensions is between 0.792 and 0.924, which is higher than 0.60 (Fornell and Larcker, 1981), indicating that the measurement structure of this study has good reliability.

**TABLE 4:** Confirmatory factor analysis.

Dimensions and items	Factor loading	CR	Cronbach’s α
**Antecedent variables (AV)**			
*Service quality*		0.823	0.898
The organizer’s timely response when encountering problems (x1)	0.712***		
Satisfaction with the organizer’s ability to resolve conflict issues (x2)	0.836***		
The organizer will take the initiative to understand the needs of exhibitors (x3)	0.672***		
The organizer provided very good on-site organization and management ability (x4)	0.782***		
*Communication*		0.872	0.879
the organizer regularly reported to the exhibitor about the exhibition (x5)	0.812***		
The exhibition organizer always informed the exhibitor of any changes related to the exhibition (x6)	0.643***		
It is Smooth communication with the organizer (x7)	0.712***		
It is frank communication between the organizer and exhibitors (x8)	0.808***		
*The effects of exhibitions*		0.792	0.875
Number of professional visitors (x9)	0.682***		
Quality of professional audience (x10)	0.713***		
Quality of exhibitors (x11)	0.756***		
Number of exhibitors (x12)	0.889***		
*Guanxi*		0.846	0.803
The consideration of a good personal relationship with the exhibition organizer (x13)	0.763***		
Choosing to participate because the exhibition is organized by the government or an industry association (x14)	0.634***		
Choosing to participate in an exhibition because they have been invited by the government or an industry association (x15)	0.641***		
**Relationship quality (RQ)**			
*Satisfaction*		0.792	0.853
Exhibitors are satisfied with the products and services provided by the organizer (y1)	0.725***		
*Trust*			
Exhibitors believe the information provided by the organizer (y2)	0.645		
**Outcome variable (OV)**			
*Exhibitors’ loyalty*		0.892	0.932
This exhibition is relatively good compared to those in which I have participated (y3)	0.715		
I will continue to participate in this exhibition in the future (y4)	0.722		
*Word of mouth*		0.924	0.952
when someone consults me, I will recommend participating in this exhibition (y5)	0.612		
I will actively recommend this exhibition to others (y6)	0.705		

*,** and *** represent the significance levels of 10%, 5% and 1%, respectively.

## Empirical analysis

### Analysis of sample basic information

The basic information of the surveyed exhibitors is shown in Table 5. Companies with a scale of fewer than 100 exhibitors accounted for the largest proportion, with a value of 49.1%. These results indicate that small and medium enterprises are the main participants in exhibitions. This result is essentially consistent with the “Report on China’s Exhibition Economic Development in 2019” issued by the CCPIT (China Council for the Promotion of International Trade). The results show that domestic sole proprietorship enterprises are the main participants in exhibitions, accounting for 81.7%. Among the companies participating in exhibitions in Beijing, more are local companies from Beijing, accounting for 65.8%. According to the data, most of the exhibitors participating in exhibitions are repeat exhibitors, thereby creating more exchanges and communication between organizers and exhibitors. Therefore, this sample is highly beneficial for studying the quality of the relationship between organizers and exhibitors. Generally speaking, 47.2% of the top leaders of the companies came to the exhibition; the proportion of middle-level leaders who led the exhibition was 45.9%, while the proportion of ordinary employees was only 6.9%.

**TABLE 5:** Basic information of survey samples.

Item option		Frequency (*N*)	Percentage (%)
Company scale	Fewer than 100 people	115	49.1
	Between 100 and 500 people	75	32.1
	More than 500 people	44	18.8
Company type	Domestic sole-source investment enterprise	188	81.7
	Joint venture	35	15.2
	Wholly foreign-owned enterprise	7	3.1
Company location	Beijing	158	65.8
	Other mainland regions	69	28.8
	Overseas	13	5.4
Exhibition experience of participants	1 time	67	28.9
	2–3 times	89	38.4
	4–5 times	30	12.9
	6 or more times	46	19.8
Highest position of exhibitors	Company senior leadership	110	47.2
	Company middle-level leaders	107	45.9
	General staff	16	6.9

### Structural equation modeling analysis results

Structural equations can deal with the path relationship between latent variables and solve the causal relationship of multiple variables simultaneously. Therefore, structural equation modeling (SEM) is widely used in the social sciences.

The theoretical analysis and model construction in Section “Methodology” demonstrate that the quality of service, communication, exhibition effectiveness, and Guanxi are four first-order latent variables that affect the quality of the relationship between exhibitors and exhibition organizers. The quality of the relationship between the two is measured by the two dimensions of satisfaction and trust, and the outcome variables caused by the quality of the relationship are two potential variables, customer loyalty and word of mouth.

#### Initial operation

This article uses AMOS 21.0 to analyze the data. The principle component was applied to conduct confirmatory factor analysis and the KMO was 0.913 and Bartlett’s Test of Sphericity was highly significant (*p* < 0.000). The main fitting indexes after analysis was conducted are shown in Table 6.

**TABLE 6:** Comparison table of fitting indexes.

Item	Absolute fit index				Value-added adaptation index		Reduced fit index	
	Significance	NC Value (ratio of chi-square degrees of freedom)	GFI	RMSEA	NFI	RFI	PNFI	CN
Adaptation standard	>0.05	1 < NC < 3	>0.90	<0.08	>0.09	>0.09	>0.05	>200
First run	0.000	3.431	0.886	0.091	0.880	0.858	0.756	251

As shown by the data in the above table, the model constructed in this research does not fit well with the data collected. Although both PNFI and CN simplified adaptation indicators have reached the adaptation standard, in terms of the absolute adaptation index, the *P* value is 0.00 < 0.05, indicating that the model rejects the null hypothesis. The NC value (the ratio of chi-square degrees of freedom) is 3.431, and neither the GFI value nor the RMSEA value reaches a good fit standard. In addition, in terms of the value-added adaptation index, the two indexes—NFI and RFI—are both less than 0.09, indicating that the model has room for improvement. Based on the judgment of the above indicators, the initial model constructed in this article needed to be modified.

After repeated adjustments and corrections, the fitting index of the model and data was finally obtained: the NC value is 2.862 < 3, GFI is 0.914 > 0.9, and RMSEA is 0.086, indicating that the adaptation is essentially reasonable; the values of NFI, RFI, and CFI are 0.924, 0.906, and 0.949, respectively, which are all greater than 0.9. The value of PNFI is 0.763 > 0.5, and the value of CN is 251 > 200. Weighing the fit of the three types of fit indicators, this article asserts that the fit between the modified model and the data is acceptable and that the model passes verification. The path coefficients between the latent variables after modification are shown in Table 7.

**TABLE 7:** Standardized test results of potential variable influence paths.

Hypothesis	Path	Estimated value γ/β	Standard error (SE)	Critical ratio (CR)	Significance	Result
Hypothesis H1a	Service quality → Relationship quality	γ_11_ = 0.38	0.036	7.012	***	Supported
Hypothesis H1b	Communication → Relationship quality	γ_12_ = 0.43	0.035	8.095	***	Supported
Hypothesis H1c	Exhibition products → Relationship quality	γ_13_ = 0.64	0.055	9.264	***	Supported
Hypothesis H1d	Guanxi → Relationship quality	γ_14_ = 0.24	0.031	6.824	***	Supported
Hypothesis H2a	Relationship quality → Customer loyalty	β_21_ = 0.89	0.069	13.910	***	Supported
Hypothesis H2b	Relationship quality → Word of mouth	β_23_ = 0–0.051	0.215	−0.236	0.814	Not supported
Hypothesis H2c	Customer loyalty → Word of mouth	β_22_ = 0.93	0.053	17.731	***	Supported

*,** and *** represent the significance levels of 10%, 5% and 1%, respectively.

**FIGURE 2:**
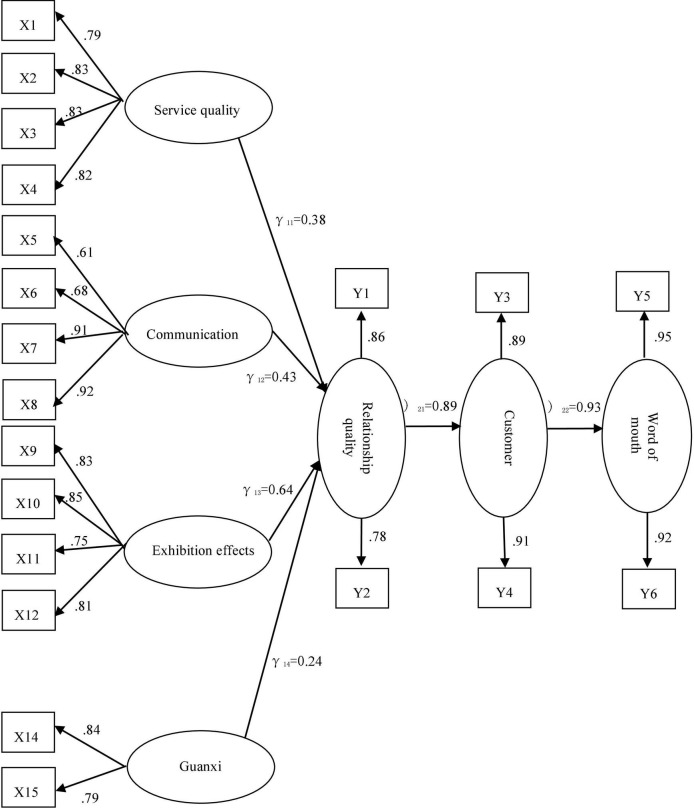
Correction diagram of the causal model of the relationship between exhibitors and exhibition organizers.

As shown in Figure 2 below, in the measurement of the relationship quality model between exhibitors and exhibition organizers, the factor loads of 21 dominant variables to the corresponding latent variables range between 0.61 and 0.95, and the minimum value is greater than the standard load value of 0.5, indicating that the observed variables offer a better interpretation of first-order latent variables. Similar to our expectations, the results of the latent variable path test in Table 7 show that the four factors of “service quality,” “communication,” “exhibitor effect” and “Guanxi” exert a significant impact on the quality of the relationship between exhibitors and exhibition organizers and that H1a, H1b, H1c, and H1d are supported. Among these results, the coefficient value of “exhibition effect” is the largest, at 0.64, indicating that if one unit increases in the exhibition effect, the quality of the relationship between organizers and exhibitors will increase by 0.64. The second is the “communication” factor, with a path coefficient of 0.43, followed by the “quality of service” and “human relations” factors, with influence coefficients of 0.38 and 0.24, respectively.

The results in Table 7 show that good relationship quality will lead to higher customer loyalty, with a path coefficient of 0.89; that is, if the relationship quality between organizers and exhibitors is increased by 1 unit, then the exhibitor’s next participation probability increases by 0.89. The influence of customer loyalty on the word-of-mouth path reached 0.93, indicating that customer loyalty exerts a significant positive influence on word of mouth. In contrast, the results also show that good relationship quality exerts no significant impact on word of mouth (*P* = 0.14 > 0.05); that is, good relationship quality between organizers and exhibitors will not prompt exhibitors to actively invite their peers to participate, so hypothesis H2b does not hold.

In summary, we will initially construct the conceptual model of this article.

### Heterogeneity test

Since the following two characteristic variables (company nature and exhibitor experience) can be divided into more than two groups, this article uses one-way analysis of variance to test the above variables. It can be seen from the results in Table 8 that the nature of the company has no significant differences in the evaluation of the pre-factor and the result factor that affect the quality of the relationship between organizers and exhibitors. Their Sig. values are all greater than 0.05. Exhibitors with rich experience. Exhibitors who participated in this exhibition with different times have evaluated the quality of the relationship between the two, and exhibitors with different annual participation times have different evaluations on the exchange factor. The Sig. values are 0.047 and 0.003 (less than 0.05) respectively. So the following focuses on the analysis of these two sets of characteristic values. We can define an exhibitor with an annual number of exhibits of 1 as an exhibitor who lacks exhibition experience, and an exhibitor with an annual number of exhibits more than 2 times as an exhibitor with rich experience in exhibiting. The data shows that compared with exhibitors who lack exhibition experience, exhibitors with rich exhibition experience have better communication effects and relationship quality with exhibition organizers. This may be related to the fact that the experienced exhibitors participate in more exhibitions each year and have had commercial contacts with various types of organizers. Therefore, they are more familiar with the exhibition process and have better communication skills with the organizers. Therefore, compared with exhibitors who lack exhibition experience, exhibitors with rich experience in participating in the exhibition are more efficient and smoother in communication with the organizer, and have a higher evaluation of the quality of the relationship between the organizers and exhibitors.

**TABLE 8:** One way analysis of variance analysis.

Factor	Enterprise nature Sig.	Participation experience Sig.
Service quality	0.258	0.313
Communication	0.390	0.003
Exhibition effect	0.403	0.150
Human relationship	0.208	0.679
Relationship quality	0.284	**0.047**
Customer loyalty	0.462	0.103
Word of mouth	0.620	0.196

The significance level is set to 0.05. The bold values means the result is significant.

## Discussion, conclusions and implications

### Discussion

The verification of the SEM structural equation confirmed the causal model of the relationship quality between organizers and exhibitors, and a model composed of 20 dominant variables and seven latent variables was obtained. “Service quality,” “communication,” “exhibition effect” and “guanxi” are the four antecedent variables of the relationship quality model, and “customer loyalty” and “word of mouth” are the outcome variables caused by the relationship quality. Compared with the conclusions of previous studies, the model constructed in this article not only covers the causal variables of relationship quality but also studies the relationship between relationship quality and the resulting variables. At the same time, this study combines the industry characteristics of the exhibition industry and the Chinese sociocultural background, discovering two new research variables: the “exhibition effect” and “guanxi.” Among them, the observation variable X13, “considering a good personal relationship with the organizer when participating in the exhibition,” was deleted from the model. This result essentially conforms to the actual situation. Exhibitors’ participation in exhibitions is a kind of business behavior. Mutual benefit and a win-win situation with the organizer is the basic condition for participation, and the pursuit of maximizing their own interests is exhibitors’ goal. Therefore, as a rational consumer, the exhibitor will not blindly participate in an exhibition because of a personal relationship with the organizer.

First, among the four antecedent variables that affect the quality of the relationship, the “exhibition effect” has the largest influence coefficient, at 0.64, indicating that exhibitors attach the highest importance to the participation effect factor. The exhibition effect includes the quality and quantity of professional visitors and the quality and quantity of exhibitors. As pointed out by Luo and Bao (2007), the quality and quantity of professional visitors are the greatest intrinsic value of an exhibition, directly affecting exhibitors’ participation decisions and overall satisfaction. The quality and quantity of exhibitors reflect the grade and reputation of an exhibition. Therefore, in the process of maintaining a high-quality relationship with exhibitors, the “exhibition effect” plays the most important role.

Second, the second-ranked contribution coefficient to the relationship quality is the “communication” factor, which has an influence value of 0.43. As we all know, exhibitions are an industry with a high degree of industrial relevance and involve all aspects of the planning and promotion of exhibition activities, venue leasing, and booth construction. Therefore, when exhibitors participate in an exhibition, they must not only communicate with the organizer to confirm the participation process but also negotiate the specific issues of booth construction and exhibit transportation with booth builders and transportation agents. The most commonly used mode of operation at present involves the organizer organizing the exhibition and inviting exhibitors to participate, with the specific operation of the exhibition and on-site service and management being completed by another or more exhibition companies, which requires exhibitors to communicate and coordinate with more parties. To ensure the smooth connection of all aspects of exhibit participation, efficient and accurate communication between the organizer and the exhibitors is extremely important.

Third, as a preimpact factor, the influence of “service quality” on relationship quality is also obvious, with a contribution value of 0.38. This conclusion is at odds with Vieira (2009) and Rauyruen and Miller (2007), who regard service quality as the outcome variable of relationship quality, and it is also slightly different from Jin et al. (2012), who use service quality as the measurement dimension of relationship quality. This article agrees with the argument of Rauyruen and Miller (2007) that good service quality will promote customer satisfaction, and only customer satisfaction will enhance the quality of the relationship between the partners. The data collected from the exhibition also supports this article’s view.

Finally, the “guanxi” factor as a newly developed influencing factor in the study of relationship quality in this article has also passed model verification, which may be related to the following two reasons: (1) Among the exhibitions held every year, government-led exhibitions account for a considerable proportion. According to research data, among the exhibitions held in Beijing in 2010, 80% of the organizers or co-organizers have backgrounds in government, industry associations or chambers of commerce, so government and industry associations exert a significant influence on exhibitions. (2) Whether it is engaging in daily life, conducting business, or making friends, Chinese people pay attention to guanxi, which is a cultural element unique to Chinese society. As Qin and Li (2020) pointed out, China is a typical ethical-based society, so Guanxi exist in all aspects of its society.

The results show that the coefficient of influence of relationship quality on exhibitors’ next exhibition is 0.89. The influence of exhibitor loyalty on word of mouth is as high as 0.93, which shows the clear importance of maintaining good customer relationships exhibition organizers. A study by the “Harvard Business Review” pointed out that the cost of a company to acquire a new customer is five times the cost of retaining an old customer, a satisfied customer will bring eight potential customers to the company, and at least one potential customer will buy the company’s products or services. Therefore, exhibition organizers must maintain loyal customers, which not only can prevent competitors from competing for this share of the market but also can bring higher profits to customers.

### Conclusion

Based on previous research results and related theories, this paper attempts to construct a causal model of the relationship quality between exhibitors and exhibition organizers. This model covers the pre- and postfactors of relationship quality. Moreover, this paper combines the local characteristics of Chinese society and culture and adds a new research dimension of relationship quality. Through field surveys of exhibitors at the Beijing Exhibition, the established causal model of relationship quality was revised, and a differentiated comparative study was conducted with exhibitors with different corporate characteristics.

First, the prefactors affecting the quality of the relationship between organizers and exhibitors are “service quality,” “communication,” “exhibition effect” and “guanxi.” These prefactors exert a significant impact on relationship quality. Among them, the “exhibition effect” exerts the most significant impact on relationship quality, followed by the “communication” factor with the organizer; the two influencing factors “service quality” and “Guanxi” have also passed the structural equation verification. Therefore, it is assumed that all the statements in H1 are supported.

Second, the quality of the relationship with the exhibition organizer exerts a positive impact on exhibitor loyalty, and exhibitor loyalty exerts a more obvious impact on exhibitors’ reputation. The effect of relationship quality on the reputation of exhibitors is not particularly significant, assuming that the conclusion part of H2 is supported.

Third, exhibitors with different corporate characteristics reflect significant differences in their evaluations of relationship quality impact factors. Exhibitors from different regions have quite different evaluations of “communication,” “Guanxi” and “customer loyalty” and “customer reputation.” Exhibitors who participated in this exhibition multiple times demonstrated significant differences in their evaluation of the quality of their relationship with the organizer, and exhibitors with different annual participation times differed in their evaluation of the “communication” factor. Therefore, it is assumed that some of the conclusions in H3 are supported.

### Implications

First, relationship quality is a key factor affecting exhibitors’ willingness to participate in the exhibition to which exhibition organizers must pay sufficient attention. With the increase of exhibitors, there is also an increasing number of exhibitions with similar themes, and in the design of specific activities, the mutual imitation and learning among exhibitors has led to a purely “differentiated” idea that cannot meet the needs of modern market competition. Thus, it is difficult for exhibitors to maintain a lasting competitive advantage within a highly competitive market space by relying solely on products and services. Whether they can establish loyal customer relationships will become one of the core factors for their success. Thus, exhibitors’ competitive strategy will shift to competition for customers to offer the best chance at success. A high-quality relationship between organizers and exhibitors can eliminate the impact of environmental changes on organizers. Therefore, organizers must regard exhibitors as a source of profits and consider customers as partners and important competitive resources for enterprises.

Second, the effectiveness of the exhibition should be improved, and exchanges and communication with exhibitors should be strengthened. The quality and quantity of professional visitors are the highest manifestations of the inherent quality of an exhibition, and the golden rule of “professional visitors win the exhibition” is once again confirmed. Therefore, as the organizer, the invitation of professional visitors should be prioritized. Exhibition organizers should abandon the long-held principle of “Exhibitor First,” which is currently the main source of income for the exhibition. Therefore, the organizer should take measures to strengthen media presence and increase the resources for professional audience invitation. The number and quality of exhibitors is also an important sign of a high-quality exhibition. The quality of exhibitors determines the reputation of an exhibition. The presence of the most well-known companies in the industry will undoubtedly improve the industry reputation of the exhibition. Other hesitant companies will also come to the exhibition based on the leading role of the “herd effect.” The number of exhibitors represents the popularity and business atmosphere of the exhibition. In addition, exhibitors’ participation fees and commercial sponsorship fees are the most important source of income for exhibitors. Therefore, the organizer should divide the exhibitors into several levels and adopt different measures for attracting exhibits according to different companies. This approach will invite strong enterprises in the industry to join, thereby improving the reputation of the exhibition and attracting more exhibitors to participate in the exhibition and thus expand its scale.

Third, the influence of Guanxi on participation decisions should be given importance, and cooperation with the government or industry associations should be strengthened. The government and industry associations play a substantial role in promoting the development of China’s exhibition industry. On the one hand, the credibility of the government or industry is higher, and exhibitors prefer and trust exhibitions with government backgrounds. On the other hand, the local government or industry associations exert a certain influence on exhibitors’ participation decisions, especially for local exhibitors, for whom the degree of such influence is more obvious. In addition, industry associations have more corporate information, and this customer information is a considerable commercial resource for the organizer. Therefore, when the exhibition organizer recruits for exhibitions and traditional sales channels, they also need to expand marketing channels and cooperate with the government or industry associations. This approach will increase the prestige of the exhibition and expand the effectiveness of exhibition recruitment and increase its income.

Finally, differentiated marketing measures should be employed for exhibitors with different corporate characteristics. Compared with local exhibitors, foreign exhibitors gave lower evaluations of factors such as “communication,” “relationship quality,” “customer loyalty” and “word of mouth” with the organizer. Therefore, the organizer should strengthen the frequency of communication with foreign exhibitors to improve the quality of such relationships, thereby increasing the probability of their repeated participation. Telephone marketing, email contact and other e-commerce methods have become the main marketing methods adopted by exhibition invitation departments. The relationship between local exhibitors and the organizer is of good quality and customer loyalty is high, so they are the key marketing objects of organizers. Exhibition organizers should make frequent greetings and even visits to consolidate the quality of their relations with exhibitors. For first-time exhibitors or those who lack exhibition experience, communication with the organizer is the main factor affecting the quality of that relationship. Therefore, the organizer must inform exhibitors of any changes to the exhibition in a timely manner and accurately convey any exhibition-related details and precautions to inexperienced exhibitors.

## Limitations and directions for future research

Although this research attempts to construct a causal model of the relationship between exhibitors and exhibition organizers, consults relevant research literature, draws on the research results of domestic and foreign scholars, and obtains some new research results, it is nonetheless limited to the author’s knowledge construction, industry cognition, research ability and research conditions. Therefore, several questions remain. The first is the rationality of the sample. Limited by objective conditions such as funding and time, this study only selected 251 exhibitors who attended one or more of four exhibitions in Beijing as the research representatives, resulting in a study with fewer subjects and a relatively concentrated time span. Therefore, the universality of the research results remains to be verified. Second is the scientific nature of the index selection. At present, research on the relationship quality between exhibitors and exhibition organizers at home and abroad is very limited. Although the author has read a large amount of relationship quality literature and combined the characteristics of the exhibition industry to establish indicators through interviews with exhibitors and consultation with experts, the indicators still need subsequent follow-up as proof of their rationality and scientific nature. Apart from this, the influence of COVID-19 has profoundly changed the way exhibitors and exhibitors participate in the exhibition. The appearance of online exhibition, electronic exhibition and other forms will have a great impact on the quality of the relationship between exhibitors and exhibitors, but the paper has not taken into account this.

Therefore, in future research, we should collect as many research representatives as possible from the exhibitions and exhibitors in first-tier exhibition cities such as Beijing, Shanghai, Guangzhou, and second-tier exhibition cities such as Chengdu, Shenzhen Dalian, and Qingdao. In addition, the time span of the investigated sample exhibitions should be broad, and the exhibition months should preferably cover the whole year. In addition, when studying the outcome variables of the relationship quality between organizers and exhibitors, the exhibitor’s exhibition brand preference should also be included in the study of relationship quality, because the influence of relationship quality on brand preference has already been confirmed in other industries such as hotels.

## Data availability statement

The original contributions presented in the study are included in the article/supplementary material, further inquiries can be directed to the corresponding author.

## Author contributions

PW contributed to the conception and design of the study. All authors contributed to the article and approved the submitted version.
